# Surgical Clip Ligation of Anterior Communicating Artery Aneurysm in a Resource-Limited Setting

**DOI:** 10.7759/cureus.14927

**Published:** 2021-05-09

**Authors:** Christopher Markosian, Igor Kurilets, Luke D Tomycz

**Affiliations:** 1 Neurological Surgery, Rutgers New Jersey Medical School, Newark, USA; 2 Neurosurgery, International Neurosurgery Center, Kyiv, UKR; 3 Neurosurgery, New Jersey Brain and Spine, Montclair, USA

**Keywords:** acom, clip, global neurosurgery, lmic, vascular

## Abstract

Anterior communicating artery (ACOM) aneurysm clipping with intraoperative measures to ensure total occlusion and avoid ischemic complications is standard in countries such as the United States. However, alternatives need to be considered in resource-limited settings. The clipping of an unruptured, superiorly projecting ACOM aneurysm in a resource-limited setting is presented and special nuances that optimize safety are described. Careful surgical technique, meticulous identification of relevant anatomy, post-ligation inspection of the aneurysm and adjacent vessels, and possibly needle puncture of the aneurysm dome are critical to achieve favorable results.

## Introduction

Neurosurgeons may need to perform surgical clip ligation of aneurysms in resource-limited settings if either (1) endovascular methods are unavailable or financially prohibitive or (2) the angio-architecture of the aneurysm lends itself more readily to open clip reconstruction (e.g., wide neck). Surgical clipping of anterior communicating artery (ACOM) aneurysms in such settings may require alternative strategies to ensure occlusion of the aneurysm and patency of the parent vessel as well as nearby perforators. While intraoperative measures are routinely used when clipping aneurysms in the United States and have been shown to make surgery safer and more efficacious [e.g., somatosensory evoked potential (SSEP)/electroencephalography (EEG) monitoring, micro-Doppler, indocyanine green (ICG) angiography, and intraoperative catheter angiography] [1], these technologies may not be available in many low- to middle-income countries (LMICs). Here, we describe the steps necessary to successfully clip an ACOM aneurysm with minimal resources based on our experience with the Co-Pilot Project in Ukraine [2]. While neuromonitoring is now being used more commonly in Ukraine, at the time of this surgery it was not available. Similarly, we did not have access to micro-Doppler, ICG angiography, or intraoperative catheter angiography at this center.

## Technical report

Relevant surgical anatomy

A standard pterional craniotomy is utilized to gain access to the aneurysm. In this case, subfrontal dynamic retraction is first used to drain cerebrospinal fluid from the perioptic cisterns and an inside-out selective splitting of the Sylvian fissure is employed to expose the relevant vascular anatomy as well as bilateral optic nerves and chiasm. Partial resection of the gyrus rectus located medially to the olfactory tract is performed for visualization of the bilateral A1/A2 segments as well as the ipsilateral artery of Heubner.

Description of the technique

**Figure 1:**
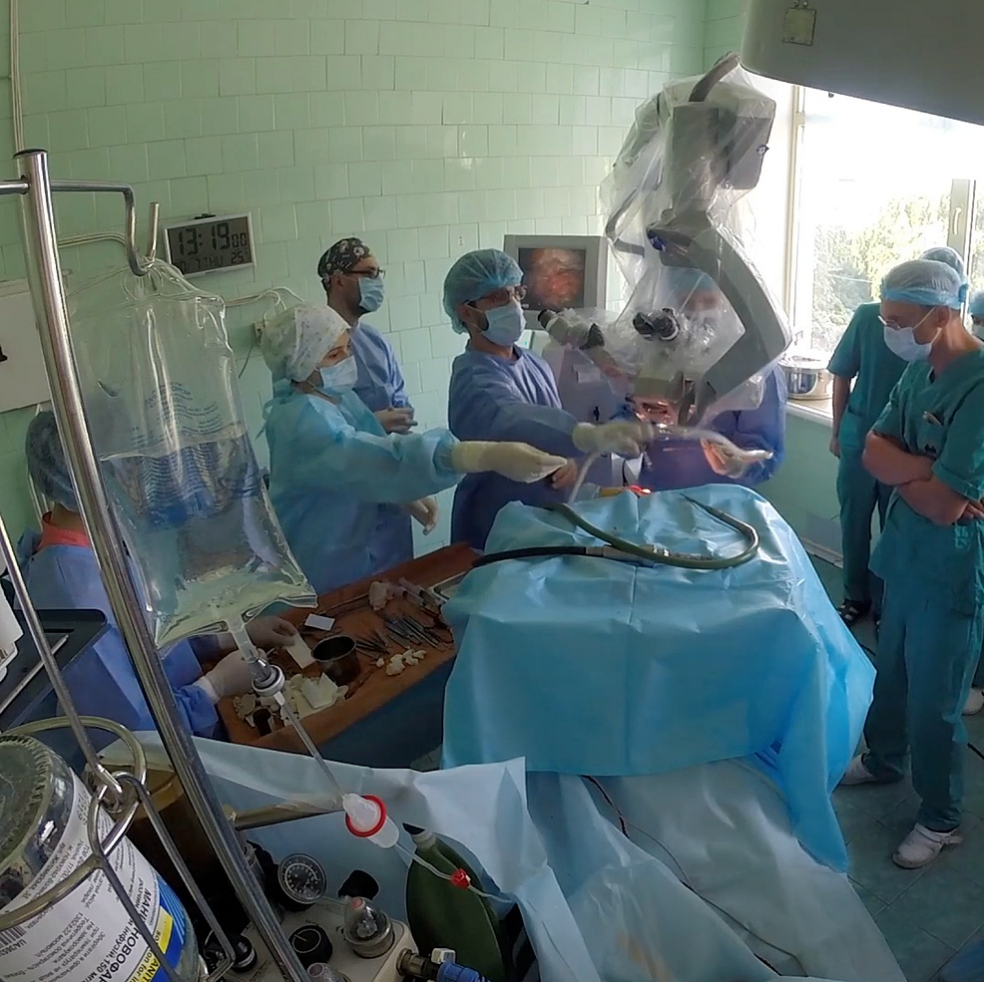
Operating room setup in a resource-limited setting for surgical clip ligation of ACOM aneurysm (Kyiv, Ukraine).

Position: The patient is positioned supine with the head pinned in a Mayfield holder (Integra LifeSciences Corporation, Princeton, NJ, USA) with extension and approximately 20° rotation, effectively positioning the malar eminence at the highest point. The patient is secured to the operating table to ensure that further rotation can be performed intraoperatively if needed. An example of an operating room setup in a resource-limited setting is depicted in Figure 1.

**Figure 2:**
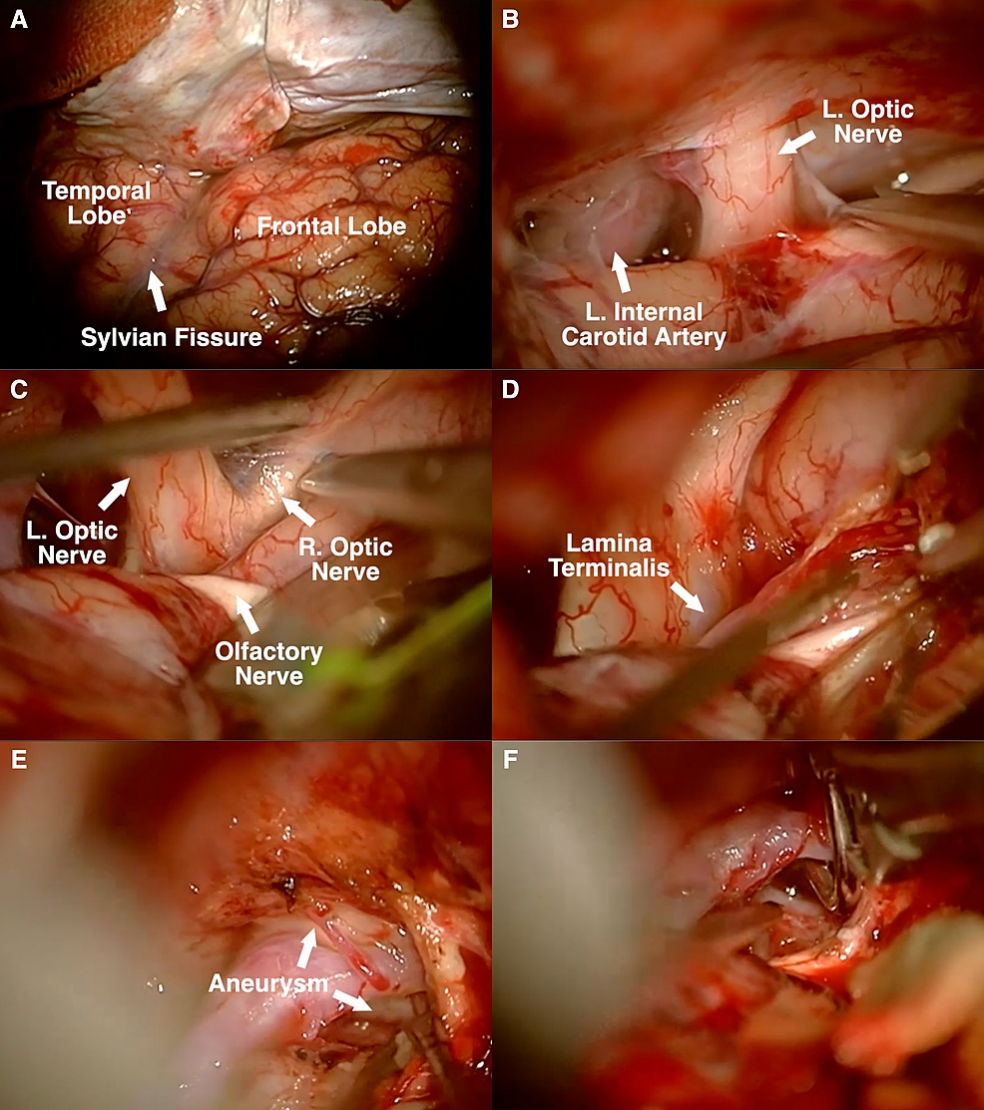
(A) Left frontotemporal (pterional) approach. (B) Subfrontal approach to expose left carotid artery and optic nerve. (C) Exposure of contralateral optic nerve. (D) Resection of gyrus rectus. (E) Early exposure of aneurysm neck. (F) Placement of fenestrated clip around ipsilateral A2.

Incision, Pterional Craniotomy, and Opening of Dura: A standard frontotemporal incision is marked extending from the root of the zygoma directly in front of the tragus of the ear toward the hairline in the midline of the head. In this case, a single musculo-fasciocutaneous flap was raised and reflected anterior-inferiorly. Craniotomy is performed using pneumatic Midas Rex drills (Medtronic, Minneapolis, MN, USA). The dura is flapped anteriorly along with the fasciocutaneous flap (Figure 2A).

Dissection of Sylvian Fissure: Extensive dissection of the Sylvian fissure is not routinely required to expose an ACOM aneurysm. However, after drilling down the sphenoid wing, one can begin with subfrontal exposure and perform a limited dissection of the deep Sylvian fissure (Figure 2B, 2C). Resection of the gyrus rectus, although not mandatory, is well tolerated and can be helpful in exposing relevant anatomy (Figure 2D).

Clipping of Aneurysm: Judicious use of a fixed retractor allows for safe clipping of the aneurysm using a 7-mm straight fenestrated clip, thereby excluding the ipsilateral A2 (Figure 2E, 2F). It is routine to identify bilateral A1s, bilateral A2s, and bilateral arteries of Heubner before definitive clipping of an ACOM aneurysm. In this case, the clip was carefully adjusted as the ipsilateral A2 appeared partially occluded after initial placement. The clip is advanced and the suction is used to manipulate the ipsilateral A2 to ensure that it remains clear of the clip blades. Retractorless surgery is becoming increasingly utilized [3], but limited and judicious use of a fixed retractor can be quite helpful and should not increase the morbidity. Indeed, in LMICs, a wide exposure for optimal visualization is recommended to maximize safety. As previously reported [4], a fenestrated clip is frequently used for a superiorly projecting ACOM aneurysm as the ipsilateral A2 typically passes over the aneurysm dome.

Post-Clipping Inspection and Puncturing of Aneurysm: While a number of adjunctive technologies are often used in the United States to ensure adequate ligation of aneurysm and patency of nearby vessels including intraoperative catheter angiography, ICG angiography, micro-Doppler, and neuromonitoring (e.g., SSEP, EEG), these technologies are not available in some resource-limited settings. Avoiding ischemic complications and ensuring adequate aneurysm occlusion requires meticulous dissection to expose relevant anatomy, identification of parent and en passage vessels and perforators, and thorough post-clip inspection of vessel patency (e.g., bilateral A1s, A2s, and arteries of Heubner). Puncture of the aneurysm dome with a needle, which is sometimes utilized to decompress neural structures, was performed in this case to confirm adequate occlusion of the aneurysm. Postoperative CT angiography can further confirm complete occlusion.

## Discussion

While there is a global trend to treat an increasing proportion of aneurysms via endovascular methods, open surgical clip ligation continues to be an essential tool in the neurovascular surgeon’s armamentarium and can be more cost-effective in certain settings [5]. Continuing to offer clip ligation may be even more critical in many LMICs where the availability and affordability of essential endovascular equipment (e.g., coils, catheters, and stents) are limited. Surgical clipping of an aneurysm in the United States is often aided by various technologies (e.g., intraoperative catheter angiography, ICG angiography, micro-Doppler, and neuromonitoring) to help assure complete ligation of the aneurysm and continued patency of the parent vessel and adjacent perforators. In the low-resource settings of many LMICs, surgeons must explore ways to safely clip aneurysms without many of these adjunctive technologies.

Our experience is based on a limited number of cases in Ukraine, but readers should be careful when trying to apply these lessons to international experiences in other countries. Risks are present with our described technique. Ischemic complications may occur if the parent vessel is clipped or perforated, which can lead to aphasia and/or hemiplegia. General risks associated with intracranial surgery include postoperative hematoma and infection. Minimizing complications is critical. First, the surgeon should be proficient in clipping techniques. Secondly, better is sometimes the enemy of good. Leaving a small aneurysmal remnant is preferable to occluding the parent vessel and causing a stroke, especially in the case of an unruptured aneurysm. Finally, adequate operative exposure, meticulous attention to detail, careful inspection of vasculature after clip placement, and needle puncture of the aneurysm dome to confirm occlusion are recommended to minimize risks.

## Conclusions

With the continued growth of neurosurgery in various LMICs and international collaborations between neurosurgeons from various countries, methods of performing operations with minimal resources may be in demand. One routine procedure in countries such as the United States is ACOM aneurysm clipping. The absence of intraoperative measures to confirm occlusion of the aneurysm and patency of the parent vessel warrants careful dissection, technical proficiency, and adequate exposure as described in this technical report.
